# Clinical evaluation of contrast-enhanced CT combined with PET/CT in diagnosis of mediastinal lymph node metastasis of non-small-cell lung cancer

**DOI:** 10.12669/pjms.38.5.5528

**Published:** 2022

**Authors:** Xiaodong Li, Xiaomeng Zheng, Tianle Zhang, Xi Dong, Jian Su

**Affiliations:** 1 Xiaodong Li, Department of Nuclear Medicine, Affiliated Hospital of Hebei University, Baoding 071000, Hebei, China; 2 Xiaomeng Zheng, Clinical Laboratory, Affiliated Hospital of Hebei University, Baoding 071000, Hebei, China; 3 Tianle Zhang, Department of Radiology, Affiliated Hospital of Hebei University, Baoding 071000, Hebei, China; 4 Xi Dong, Department of Nuclear Medicine, Affiliated Hospital of Hebei University, Baoding 071000, Hebei, China; 5 Jian Su, Department of Nuclear Medicine, Affiliated Hospital of Hebei University, Baoding 071000, Hebei, China

**Keywords:** Contrast-enhanced CT, PET/CT, Non-small-cell lung cancer, Mediastinal lymph node metastasis, Differential diagnosis

## Abstract

**Objectives:**

To investigate the clinical value of contrast-enhanced CT combined with PET/CT in the differential diagnosis of mediastinal lymph node metastasis (MLNM) of non-small-cell lung cancer (NSCLC).

**Methods:**

A total of 120 patients with NSCLC combined with mediastinal lymphadenopathy hospitalized in our hospital were selected. All the patients received radical resection of lung cancer and mediastinal lymphadenectomy. After pathological diagnosis, they were divided into MLNM group (malignant group, undergoing contrast-enhanced CT) and non-MLNM group (benign group, receiving contrast-enhanced CT combined with PET-CT). The results were judged by two senior radiologists independently. The results of different scanning methods and postoperative pathology were compared using the t test, χ^2^ test and Pearson correlation coefficient test.

**Results:**

Compared with the pathological results, contrast-enhanced CT diagnosed 31 cases, with a coincidence rate of 62%, and contrast-enhanced CT combined with PET-CT diagnosed 42 cases, with a coincidence rate of 84%, presenting a statistically significant difference (*P* = 0.02). Among the 120 patients with lung cancer, pathological examination confirmed MLNM in 50 patients and benign enlargement in 70 patients, contrast-enhanced CT alone detected metastasis in 40 patients and benign enlargement in 80 patients, and contrast-enhanced CT combined with PET-CT detected metastasis in 47 patients and benign enlargement in 73 patients. The sensitivity and accuracy of the latter were significantly higher than those of the former (sensitivity, *P* = 0.01; accuracy, *P* = 0.01). With the increase in the malignancy of lymph nodes, the degree of CT enhancement, the concentration of radioactive substances and SUV value increased, showing positive correlations.

**Conclusion:**

Contrast-enhanced CT combined with PET/CT in the diagnosis of MLNM of NSCLC presents higher coincidence rate, sensitivity and accuracy. With the increase in tumor malignancy, the enhancement degree and radioactive substance concentration increase. The two methods are synergistic and complementary in diagnosing MLNM.

## INTRODUCTION

At present, lung cancer is one of the malignant tumors with the highest incidence and fatality in the world^1^, which is a great threat to human life and health. Non-small-cell lung cancer (NSCLC) is the most common type of lung cancer, accounting for about 85% of all pathological types of lung cancer.^2^ Surgery is the main treatment method for NSCLC.^3^ The evaluation of regional lymph node metastasis is vital for the selection of treatment method, and also an important factor affecting the prognosis of patients.^4^ For the preoperative evaluation of primary lesions and metastases, the most commonly used methods include CT and PET-CT.^5^ CT has a certain advantage in the diagnosis of primary lesions, but it is less sensitive for small metastases.^6^ PET-CT imaging can reflect not only the anatomical information of lesions, but also the biological activity of tumor lesions, so PET-CT is the gold standard to determine the tumor staging of lung cancer.^7^ In addition, PET-CT also has a superiority in the diagnosis of lymph node metastasis of lung cancer.^8^ Current studies are more inclined to CT and PET-CT in the evaluation of primary lesions, but rarely investigate mediastinal lymph nodes. Therefore, we evaluated the mediastinal lymph nodes of NSCLC using contrast-enhanced CT and contrast-enhanced CT combined with PET/CT.

## METHODS

A total of 120 patients with NSCLC combined with mediastinal lymphadenopathy hospitalized in our hospital were selected. All the patients received radical resection of lung cancer and mediastinal lymphadenectomy. After pathological diagnosis, they were divided into MLNM group (malignant group, n = 50) and non-MLNM group (benign group, n = 70). The malignant group included 33 males and 17 females, aging 58-75 years (average, 63.54 ± 7.28 years). In the benign group, there were 51 males and 19 females, with an age of 55-73 years (average, 62.31 ± 6.74 years). The general data (gender and age) showed no significant differences between the two groups (*P* > 0.05), suggesting comparability. The diameter of primary lesions was 3.40 ± 0.85 cm in the malignant group and 2.76 ± 0.32 cm in the benign group, presenting a larger diameter in the malignant group (*P* = 0.00) (Table-I).

**Table I:** Comparison of general data between the two groups (χ¯± S).

Index	Malignant group(50)	Benign group(70)	t/χ^2^	*P*
Age (years)Δ	63.54±7.28	62.31±6.74	0.95	0.34
Male (N %)Δ	33(66%)	51(72.8%)	0.65	0.42
Diameter of primary lesions (cm)*	3.40±0.85	2.76±0.32	5.76	0.00
Pathological typeΔ				
Adenocarcinoma	15(30%)	22(31.4%)	0.13	0.91
Squamous cell carcinoma	28(56%)	35(50%)	0.24	0.62
Others	7(14%)	13(18.6%)	0.44	0.51
Diameter of mediastinal lymph nodes (cm) Δ	1.24±0.12	1.21±0.15	0.78	0.44

Δp > 0.05,

* p < 0.05.

### Ethical approval:

The study was approved by the Institutional Ethics Committee of Affiliated Hospital of Hebei University, (Date May 27, 2021) and written informed consent was obtained from all participants.

### Inclusion criteria:

- Patients diagnosed as NSCLC^9^ and mediastinal lymphadenopathy;
- Patients receiving radical resection of lung cancer and mediastinal lymphadenectomy;
- Patients undergoing chest CT or PET-CT within 1 month before surgery, with complete imaging data;
- Patients without chemoradiotherapy before admission;
- Patients with definite lesions detected by chest imaging examination (CT or MRI), and the size of lesions accurately evaluated^10^;
- Patients with clear consciousness and no mental illness;
- Patients and their families with willingness and ability to cooperate to complete the study, and good treatment compliance.

### Exclusion criteria:

- Patients with metastatic lung cancer or lung cancer of other pathological types;
- Patients without mediastinal lymphadenectomy or effectively evaluated nature of mediastinal lymph nodes;
- patients with incomplete clinical data;
- patients combined with severe cardiopulmonary diseases;
- patients with mental illness or other cognitive impairment, and no ability to cooperate the study;
- patients combined with malignant tumors in other parts.

### Scanning method:

In the supine position, fasting patients received scanning using Philips 128-slice spiral CT, with scanning parameters as follows: tube current, 50-300 mAs; tube voltage, 120 kV; spiral pitch, 0.984; rotation time, 0.4 s/revolution; noise figure, 10-11; slice thickness, 1.25 mm. All the subjects completed the scanning by holding their breath at the end of inspiration, ranging from the apex to the bottom of the lung. Ioversol (350 mg I/ml, 1.2 ml/kg) was injected through the peripheral vein at a flow rate of 3.0 ml/s. After the injection of contrast medium, scanning was performed 28 s and 45 s later, respectively.

### Result judgment:

The original images were uploaded to the GE AW 4.7 post-processing station, and all the data were analyzed and processed using GSI Viewer. The homogeneous parts of the parenchymal lesions were selected as regions of interest (ROIs), and the energy spectrum curve was measured. The slope of the curve was calculated by two or more physicians above deputy director. According to the slope of the curve, the lymph nodes were preliminarily diagnosed: the slope difference between primary lesions and lymph nodes < 0.2, metastatic; > 0.2, non-metastatic.^11^

PET-CT scanning was conducted using Philips Vereos PET/CT scanner. After fasting for more than six hour, 0.12 mCi/kg ^18^F-FDG was injected intravenously. After resting for 60 minutes, whole-body PET-CT was performed in three-dimensional mode. PET-CT images were reconstructed by filtered back projection. After data acquisition, the data were transferred to the workstation of the system for image fusion, and the reconstructed images were collected and the SUV value of the lesions was obtained. Then, diagnosis was made through SUV measurement and visual inspection by two physicians above deputy director of nuclear medicine. SUV measurement results: normal: SUV value < 2.0; malignant: SUV value > 2.5; benign: SUV value = 2.0-2.5. Visual inspection results: lymph nodes without radioactive concentration were considered as benign lesions; lymph nodes with radioactive concentration were considered as malignant or metastatic lesions.

### Observation indicators:

The coincidence rate of different examination methods with pathological results was compared. The sensitivity, specificity and accuracy of contrast-enhanced CT alone and contrast-enhanced CT combined with PET/CT in the diagnosis of MLNM were compared. The correlations of mediastinal lymph nodes with different natures with CT enhancement and PET/CT radioactive concentration were compared.

### Statistical Analysis:

All data were statistically analyzed using SPSS 20.0. The measurement data were expressed as (X̄ ± S). Two independent samples t-test was used to analyze the data between groups, and χ^2^ test was used to compare the rates. The correlation was expressed by Pearson correlation coefficient. *P* < 0.05 was considered as statistically significant.

## RESULTS

The comparison of different examination methods with the pathological results (Table-II) showed that among the 50 patients with MLNM, contrast-enhanced CT diagnosed 31 cases, with a coincidence rate of 62%, and contrast-enhanced CT combined with PET-CT diagnosed 42 cases, with a coincidence rate of 84%. The coincidence rate had a statistically significant different between the two methods (*P*= 0.02).

**Table II:** Comparison in coincidence rate of different methods with malignancy (MLNM) (χ¯± S) n = 50.

Group	Malignancy	Pathological diagnosis	Coincidence rate*
Contrast-enhanced CT	31	50	62%
Contrast-enhanced CT combined with PET-CT	42	50	84%
χ^2^			6.14
*p*			0.02

* *P* < 0.05.

Among the 120 patients with lung cancer, pathological examination confirmed MLNM in 50 patients and benign enlargement in 70 patients, contrast-enhanced CT alone detected metastasis in 40 patients and benign enlargement in 80 patients, and contrast-enhanced CT combined with PET-CT detected metastasis in 47 patients and benign enlargement in 73 patients. The sensitivity and accuracy of the latter were significantly higher than those of the former (sensitivity, *P* = 0.01; accuracy, *P* = 0.01), as seen in Table-III & IV.

**Table III:** Correlations of contrast-enhanced CT alone and contrast-enhanced CT combined with PET/CT with pathological results.

Pathological results	Contrast-enhanced CT alone			Contrast-enhanced CT combined with PET/CT		

	Malignant	Benign	Total	Malignant	Benign	Total
Malignant	31	19	50	42	8	50
Benign	9	61	70	5	65	70
Total	40	80	120	47	73	120

**Table IV:** Analysis of diagnostic sensitivity, specificity and accuracy of contrast-enhanced CT alone and contrast-enhanced CT combined with PET/CT.

Group	Sensitivity*	Specificity	Accuracy*
Contrast-enhanced CT	62% (31/50)	87% (61/70)	76.6% (92/120)
Contrast-enhanced CT combined with PET-CT	84%(42/50)	92%(65/70)	89.2% (107/120)
χ^2^	6.14	1.27	6.62
P	0.01	0.26	0.01

* *P* < 0.05

The correlation analysis between nature of mediastinal lymphadenopathy and CT enhancement and PET/CT radioactive concentration demonstrated that with the increase in the malignancy of lymph nodes, the degree of CT enhancement, the concentration of radioactive substances and SUV value increased, showing positive correlations, indicating that contrast-enhanced CT and PET-CT have a synergistic effect in judging the nature of enlarged lymph nodes.Table-V

**Table V:** Correlations between nature of mediastinal lymphadenopathy and CT enhancement and PET/CT radioactive concentration.

Nature of lymph nodes	CT enhancement	Radioactive concentration	SUV value
Malignant tumor	0.36	0.43	0.37
Benign enlargement	0.33	0.35	0.31
Normal lymph nodes	0.30	0.27	0.24

## DISCUSSION

Lung cancer is the most common malignant tumor of the respiratory system, among which NSCLC is the most common. For patients with NSCLC, the main treatment method is surgery-based comprehensive treatment.^12^ Its prognosis is closely related to the timing of treatment, early detection and timely treatment can present a satisfactory effect.^13^ It is believed that^14^ NSCLC with different genotypes have different tendencies of lymph node metastasis, which has a great influence on the selection of treatment methods. Yang et al.^15^ believe that patients with lymph node metastasis and multiple-organ metastasis present poorer prognosis in terms of survival time, and a better understanding of lymph node metastasis can help clinicians better select treatment methods for NSCLC.

Mediastinal lymph node is a common metastatic site of NSCLC. The number and area of MLNM are closely related to the surgical method, resection range and postoperative recurrence.^16^ At present, CT, bronchoscopy, mediastinoscopy and thoracoscopy are the methods for clinical diagnosis of MLNM. However, invasive surgery is not easily accepted by patients before definite diagnosis. With the rapid development of CT technology, it has gradually become an imaging method for noninvasive diagnosis of the pathological staging of NSCLC.^17^ Nevertheless, the value of contrast-enhanced CT in the diagnosis of lymph node metastasis is limited. CT value reflects the attenuation ability of substances to X-ray, but conventional CT can not accurately reflect the attenuation characteristics of substances to X-ray, which is more obvious in small lesions.^18^

PET-CT is a newly developed scanning method in recent years. In clinic, ^18^F-FDG, as a deoxyglucose analogue, is often used as a PEF-labeled nuclide. After entering the human body, it can be rapidly transported across the cell membrane by glucose transporters and enter the cell fluid, and then is transformed into F-FDG-6-PO4 by the phosphorylation of hexokinases in the cell fluid. The latter can not be recognized by fructokinase-1 and is catalysed for further metabolic process, so it can be retained in histocytes for a long time for developing.^19^ Compared with normal tissues, F-FDG is highly absorbed by human tumor tissues, presenting obviously local development, which can be used for early detection and diagnosis of malignant tumors. It has been pointed out that PET-CT scanning has higher detection rate and more significant application value in the diagnosis of lung cancer and lymph node metastasis.^20^

However, Wang et al.^21^ believe that the sensitivity and specificity of PET-CT for MLNM need further prospective evaluation. Moreover, invasive surgery and pathological confirmation are needed.^22^,^23^ A meta-analysis^24^ has found that PET/CT has moderate sensitivity and specificity in predicting MLNM in patients with NSCLC, and it may not be used to predict or exclude lymph node metastasis in patients with NSCLC.

Additionally, the study of Bustos^25^ suggested that ^18^F-FDG PET-CT had a sensitivity of 53.8%, a specificity of 76.6%, a positive predictive value of 38.9%, a negative predictive value of 85.7%, and an accuracy of 71.7% in diagnosing MLNM. Multivariate analysis showed that the factors related to false negative results were moderate differentiation (*P* = 0.005) and SUVmax > 4 of primary tumors (*P* = 0.027). Considering the high false positive rate, it is suggested that the positive cases should be confirmed by histology.

Contrast-enhanced CT combined with PET/CT has a complementary effect in determining the malignancy of lesions^26^, and can improve the diagnostic accuracy. Our study confirmed that with the increase in the malignancy of lymph nodes, the degree of CT enhancement, the concentration of radioactive substances and SUV value all increased, showing positive correlations. Compared with the postoperative pathological results, the coincidence rate of CT alone and CT combined with PET-CT was 62% and 84%, respectively, with a statistically significant difference (*P* = 0.02). The positive rate of MLNM was 75.5% in patients with primary lesions > 3 cm, and 41.2% in patients with primary lesions < 3 cm. Patients with primary lesions > 3 cm were more prone to MLNM (*P* = 0.00), but there was no significant correlation between MLNM and lymph node size (*P* = 0.44). The sensitivity and accuracy were 62% and 76.6% in contrast-enhanced CT alone, and 84% and 89.2% in contrast-enhanced CT combined with PET-CT, respectively, showing statistically significant differences (sensitivity, *P* = 0.01; accuracy, *P* = 0.01). Furthermore, the diagnostic sensitivity and accuracy of contrast-enhanced CT combined with PET-CT were significantly higher than those of PET-CT alone reported in literature.

### Limitation of this study:

It includes small sample size. Retrospective study of patients with mediastinal lymphadenopathy was carried out, without stricter N staging and more rigorous analysis according to N staging. We are further collecting cases and clinical data, and carried out a prospective analysis of some patients, in the expectation to early and accurate assessment of patients, so that more patients benefit.

## CONCLUSION

Contrast-enhanced CT combined with PET/CT in the diagnosis of MLNM of NSCLC presents higher coincidence rate, sensitivity and accuracy. With the increase in tumor malignancy, the enhancement degree and radioactive substance concentration increase. The two methods are synergistic and complementary in diagnosing MLNM.

### Authors’ Contributions:

**XL & XZ:** Designed this study and prepared this manuscript, are responsible and accountable for the accuracy and integrity of the work.

**TZ & XD:** Collected and analyzed clinical data.

**JS:** Significantly revised this manuscript.
